# Improvement of long-term clinical outcomes by successful PCI in the very elderly women with ACS

**DOI:** 10.1186/s12872-021-01933-7

**Published:** 2021-03-04

**Authors:** Jia-li Wang, Chun-yan Guo, Hui Chen, Hong-wei Li, Xue-qiao Zhao, Shu-mei Zhao

**Affiliations:** 1 Department of Cardiology, Cardiovascular Center, Beijing Friendship Hospital, Capital Medical University, No.95, Yongan Road, Xicheng District, Beijing, 100050 People’s Republic of China; 2 Department of Internal Medical, Medical Health Center, Beijing Friendship Hospital, Capital Medical University, Beijing, 100050 People’s Republic of China; 3 Beijing Key Laboratory of Metabolic Disorder Related Cardiovascular Disease, Beijing, 100069 People’s Republic of China; 4 Division of Cardiology, University of Washington, Seattle, WA 98104 USA

**Keywords:** Very elderly female patients, Acute coronary syndromes (ACS), Percutaneous coronary intervention (PCI), Major adverse cardiovascular and cerebrovascular event (MACCE), Prognostic factors

## Abstract

**Background:**

Whether very elderly women with acute coronary syndromes (ACS) should receive aggressive percutaneous coronary intervention (PCI) is still controversial. We assessed the effectiveness and long-term clinical outcomes of successful PCI in this population and identified prognostic factors which might contribute to the incidence of major adverse cardiovascular and cerebrovascular events (MACCE) in the very elderly female PCI cohort.

**Methods:**

Female ACS patients aged ≥ 80 years were consecutively enrolled (n = 729) into the study. All the patients were divided into female PCI group (n = 232) and medical group (n = 497). MACCE was followed up, including non-fatal myocardial infarction (MI), stroke, heart failure requiring hospitalization (HFRH), cardiovascular (CV) death, and the composite of them. After propensity score matching (1:1), the incidences of MACCE were compared between the two groups. Clinical and coronary artery lesion characteristics were compared between the female PCI patients with (n = 56) and without MACCE (n = 176). Multivariate Cox regression analysis was performed to identify risk factors which independently associated with MACCE in the female PCI patients. MACCE of male PCI patients, who aged ≥ 80 years and hospitalized in the same period (n = 264), was also compared with that of the female PCI patients.

**Results:**

A total of 32% very elderly female ACS patients received PCI in the present study. (1) Compared to female medical group, PCI procedure significantly alleviated the risks of MACCE: non-fatal MI (6.2% vs. 20.2%, *P* < 0.001), HFRH (10.9% vs. 22.5%, *P* = 0.012), CV death (12.4% vs. 28.7%, *P* < 0.001) and the composite MACCE (24.0% vs. 44.2%, *P* < 0.001) during the median follow-up period of 36 months. (2) Between very elderly female and male PCI patients, there were no significant differences in occurrence of MACCE (*P* = 0.232) and CV death (*P* = 0.951). (3) Multivariate Cox analysis revealed that ST-segment elevation myocardial infarction (STEMI) (HR 1.944, 95% CI 1.11–3.403, *P* = 0.02) and elevated log- N-Terminal pro-brain natriuretic peptide (NT-proBNP) (HR 1.689, 95% CI 1.029–2.773, *P* = 0.038) were independently associated with the incidence of MACCE in the female PCI patients.

**Conclusions:**

PCI procedure significantly attenuated the risk of MACCE and improved the long-term clinical outcomes in very elderly female ACS patients. Aggressive PCI strategy may be reasonable in this population.

## Background

Acute coronary syndrome (ACS) has been the leading cause of death worldwide [1, 2]. The extensive application of percutaneous coronary intervention (PCI) has significantly improved the clinical outcomes of ACS patients [3, 4]. However, there are still controversies and concerns about PCI treatment in very elderly female patients with ACS [5, 6]. Very elderly women are a special cohort with their own clinical characteristics, which may affect the clinical outcomes of PCI in this population. For example, very elderly women with ACS were detected to have more risk factors or comorbidities, such as hypertension, diabetes and cerebrovascular disease [7], higher bleeding complications when treated with dual antiplatelet therapy (DAPT) [8, 9], and more severe coronary artery lesions [10, 11].

In clinical practice, cardiologists are more likely to avoid PCI procedure for very elderly women because of the potential higher risk of complications. Limited studies had also revealed that very elderly female ACS patients had received significantly less invasive angiography and timely revascularization [12, 13] for higher mortality rates [12, 14]. Furthermore, very elderly women treated with PCI for ACS were often excluded from large international clinical trials [15, 16]. As a result, there are less clinical studies and clinical experiences of PCI procedure in this population. The aims of the present study were to investigate the effectiveness and clinical outcomes of PCI procedure in the very elderly female ACS patients in the era of drug-eluting stents, and to identify the possible risk factors which contributed to the incidence of MACCE in the very elderly female PCI cohort.

## Methods

### Study population and protocol

A total of 1449 ACS patients aged 80 years or older, who were admitted to Cardiovascular center, Beijing Friendship Hospital between January 2013, and December 2018, were enrolled into the study. The medical retrospective data were recruited and recorded in CBD-BANK (Cardiovascular Center Beijing Friendship Hospital Database Bank) supported by DHC SOFTWARE system (DongHua company SOFTWARE CO., LTD). ACS, including ST-elevation myocardial infarction (STEMI), non-ST-elevation MI (NSTEMI), and unstable angina pectoris (UAP), were diagnosed, and confirmed based on symptoms, electrocardiogram (ECG) changes, cardiac biomarkers. The treatment options of PCI or medication only depended on the patients’ clinical situation, and decisions were made by two cardiologists simultaneously according to the international standards and guideline [17].

**Fig. 1:**
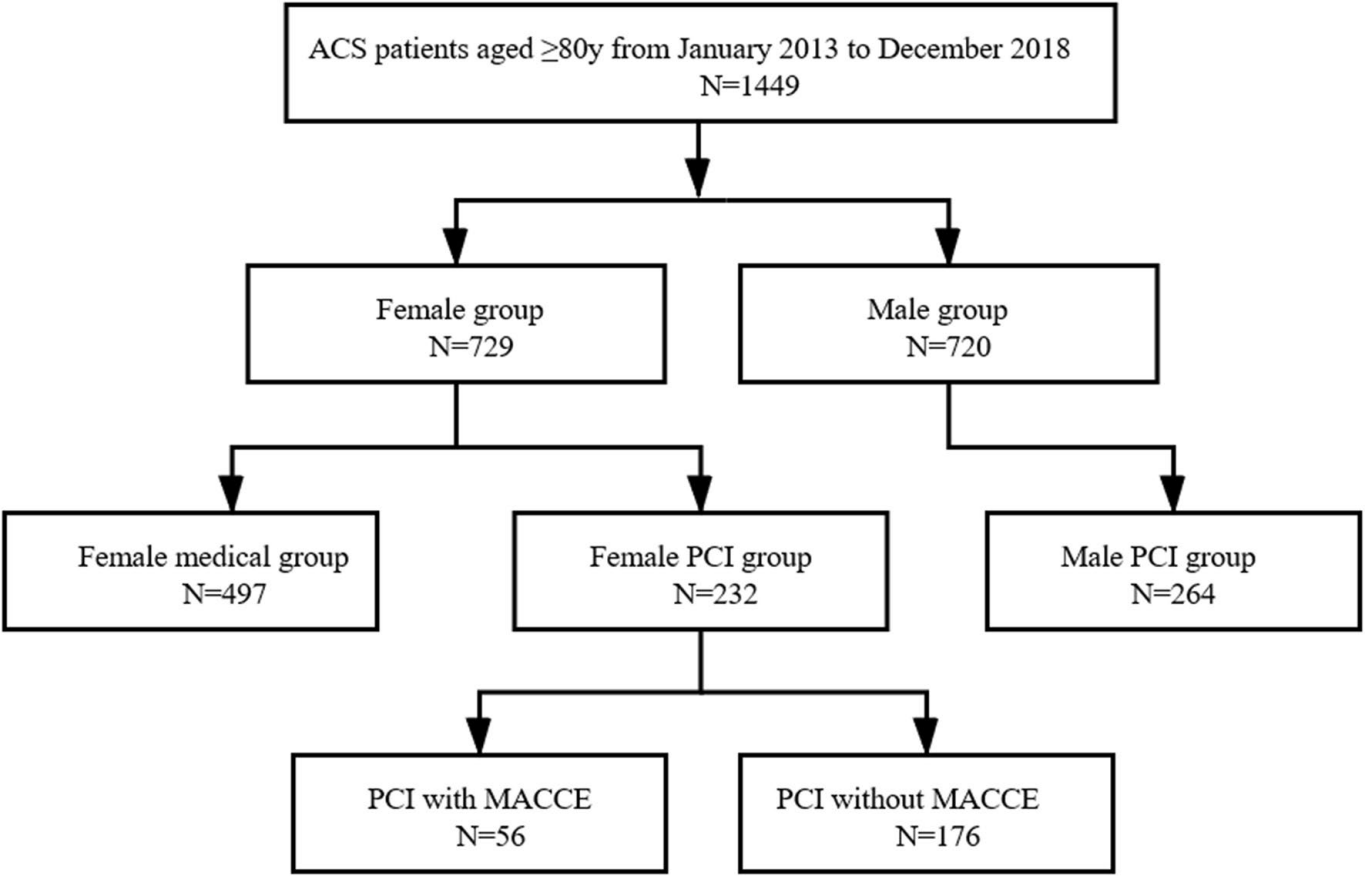
Patients enrollment flow. *ACS* acute coronary syndrome, *PCI* percutaneous coronary intervention, *MACCE* major adverse cardiovascular and cerebrovascular events

Study protocol was described as follow (Fig. 1): (1) Consecutive ACS patients aged ≥ 80 years old (n = 1449) were enrolled. (2) All the patients were classified by gender: female group (n = 729), and male group (n = 720). (3) Within the female group, patients were categorized into the PCI group (n = 232) and the medical group (n = 497). (4) Between the female PCI group and medical group, clinical characteristics, and follow-up MACCE were recorded and compared before and after Propensity score (PS) matching (1:1). (5) Within female PCI group, clinical and coronary artery characteristics were analyzed and compared in female PCI patients with (n = 56) and without MACCE (n = 176). Independent prognostic factors for the incidence of MACCE were detected in the female PCI patients. (6) MACCE of male PCI patients, aged ≥ 80 years old and hospitalized in the same period (n = 264), was also compared with the female PCI patients. The study protocol was approved by Institutional Ethics Committee of Beijing Friendship Hospital, and the study was also in accordance with the 1964 Declaration of Helsinki and its later amendments or comparable ethical standards.

### Data collection

Baseline data were collected and analyzed, including demographic information, initial clinical presentation (heart rate, systolic/diastolic blood pressure at admission), and past medical history (hypertension, diabetes, dyslipidemia, prior MI/stroke and smoking). Laboratory examination results involved hemoglobin A1c, lipid spectrum, creatinine, alanine aminotransferase (ALT), N-Terminal pro-brain natriuretic peptide (NT-proBNP), and creatine kinase-MB (CK-MB), measured during hospitalization. M-mode and two-dimensional echocardiography (ECHO) were performed (Philips IE33) for routine parameters, such as left ventricular ejection fraction (LVEF). Characteristics of coronary artery lesion and stent implantation information were evaluated by angiographic and PCI procedure, presenting in the medical documents. Severe coronary artery stenosis was defined as epicardial main coronary artery stenosis ≥ 75%  [18]. Chronic total occlusion (CTO) referred to 100% coronary artery occlusion with TIMI grade 0 flow and more than three months [19]. Successful PCI was identified as the attainment of a residual vessel diameter stenosis ≤ 20% and normal epicardial coronary flow (TIMI-3 flow)  [20]. Hemorrhage was confirmed as “major” if there was a reduction of hemoglobin ≥ 5 g/dL or any intracranial bleeding  [21]. Medication after discharge was determined from the medical records or regular telephone follow-up.

### Primary and secondary endpoints and follow-up

Composite MACCE was defined as the primary endpoint, which was the combination of cardiovascular (CV) death, non-fatal MI, stroke, and heart failure requiring hospitalization (HFRH). The secondary endpoints included all-cause death and each of the adverse events mentioned above. CV death was referred to death from MI, HF, or documented cardiac sudden death. Stroke was new ischemic stroke that occurred during follow-up, confirmed by symptoms, results of computed tomography scan. Non-fatal MI and HFRH were determined by the information from medical records, including symptom, physical sign, results of ECG and ECHO, and the value of biomarkers. All MACCE were confirmed by two separate cardiologists simultaneously. Regular follow-up was conducted by clinic visits or phone interviews every 1–3 months until Dec 2019.

### Statistical analysis

Continuous variables were expressed as median with interquartile range and were compared by Mann–Whitney U test. Categorical data were expressed as frequencies or percentages and were compared by Chi-square or Fisher’s exact statistics. Propensity score matching (1:1) was performed between female PCI group and medical group to reduce the effect of treatment-selection bias and potential confounding factors in the present study.

The primary and secondary endpoints were compared between female PCI and medical groups, as well as female PCI group and male PCI group (≥ 80 years) hospitalized at the same period by Chi-square test. Survival curves were conducted by Kaplan–Meier method and compared with the log-rank test. Univariable and multivariable Cox proportional hazards analysis were used to estimate the hazard ratios (HR) and 95% confidence intervals (CI) for composite MACCE in the very elderly female PCI cohort. Based on possible confounding variables identified from the univariate analysis, multivariable Cox regression models in an all-enter way were constructed to determine the independent predictors for the incidence of composite MACCE in the female PCI group.

A two-sided *P* value < 0.05 was statistically significant. All the statistical analysis was conducted by the SPSS software version 23.0 (IBM, Armonk, NY, USA).

## Results

### Clinical characteristics at baseline

Of 729 very elderly female ACS patients enrolled, the mean age was 82 (IQR 81, 84) years, 32% (n = 232) received PCI treatment, and 68% (n = 497) received medical treatment only. Among them, 22% received primary PCI (n = 52) and 78% was selective PCI (n = 180). Clinical characteristics of the study cohort were described in Table 1. 62% (n = 454) cases had UAP, 22% (n = 159) had NSTEMI and 16% (n = 116) had STEMI.

**Table 1:** Clinical characteristics for PCI and medical group in very elderly female ACS patients

Variables	Before PS match		*P* value	After PS match		*P* value
	PCI group (n = 232)	Medical group (n = 497)		PCI group (n = 129)	Medical group (n = 129)	
Demographic						
Age (years)	82 (81, 84)	82 (81, 85)	0.082	82 (81, 84)	82 (81, 85)	0.273
BMI (kg/m^2^)	24.4 (22.3, 27.0)	24.4 (22.0, 26.9	0.6	24.4 (22.6, 27.3)	24.0 (21.9, 27.3)	0.327
Initial presentation						
Heart rate (beats/min)	70 (65, 79)	70 (63, 81)	0.961	70 (64, 76)	72 (65, 84)	0.057
Systolic BP (mmHg)	134 (120, 149)	133 (122, 147)	0.782	135 (124, 149)	130 (120, 148)	0.168
Diastolic BP (mmHg)	70 (62, 79)	70 (64, 80)	0.371	70 (63, 77)	70 (64, 80)	0.733
Past medical history						
Hypertension, n (%)	185 (79.9)	422 (84.9)	0.082	102 (79.1)	108 (83.7)	0.337
Diabetes mellitus, n (%)	92 (39.7)	175 (35.2)	0.246	48 (37.2)	49 (38.0)	0.898
Dyslipidemia, n (%)	96 (41.4)	216 (43.5)	0.597	54 (41.9)	44 (34.1)	0.20
Prior MI, n (%)	17 (7.3)	51 (10.3)	0.202	9 (7.0)	9 (7.0)	0.986
Prior stroke, n (%)	56 (24.1)	137 (27.6)	0.329	30 (23.3)	31 (24.0)	0.884
Smoking, n (%)	23 (9.9)	29 (5.8)	*0.046*	8 (6.2)	10 (7.8)	0.625
Prior PAD, n (%)	2 (1.0)	3 (0.7)	0.688	0 (0)	3 (2.4)	0.247
Clinical diagnosis						
UAP, n (%)	111 (47.8)	343 (69.0)	< *0.001*	66 (51.2)	58 (45.0)	0.319
NSTEMI, n (%)	55 (23.7)	104 (20.9)	0.397	41 (31.8)	42 (32.6)	0.894
STEMI, n (%)	66 (28.4)	50 (10.1)	< *0.001*	22 (17.1)	29 (22.5)	0.274
Length of stay (days)	7 (6, 10)	7 (6, 10)	0.461	7 (6, 10)	8 (6, 11)	0.173
Laboratory finding						
HbA1c (%)	6.2 (5.7, 7.2)	6.1 (5.7, 6.9)	0.30	6.3 (5.7, 7.2)	6.2 (5.7, 7.0)	0.753
TC (mmol/L)	4.41 (3.69, 5.26)	4.26 (3.60, 5.02)	0.087	4.4 (3.70, 5.1)	4.3 (3.6, 5.2)	0.683
LDL-C (mmol/L)	2.46 (1.99, 3.03)	2.34 (1.81, 2.89)	*0.012*	2.49 (1.96, 2.99)	2.43 (1.91, 2.97)	0.510
TG (mmol/L)	1.32 (0.96, 1.85)	1.24 (0.90, 1.67)	0.052	1.32 (0.98, 1.84)	1.22 (0.89, 1.67)	0.183
Creatinine (umol/L)	76.3 (65.9, 91.7)	79.5 (67.9, 100.2)	*0.016*	79 (66.6, 95.7)	81.6 (68.4, 99.1)	0.299
ALT (U/L)	15 (10, 21.8)	13 (10, 20)	*0.024*	15 (11, 21)	14 (10, 22)	0.586
CK-MB (ng/ml)	1.7 (1.1, 4.05)	1.4 (0.9, 2.53)	*0.002*	1.7 (1.1, 4.5)	1.8 (1.08, 5.95)	0.553
log NT-proBNP	2.93 (2.56, 3.42)	3.05 (2.49, 3.58)	0.231	2.92 (2.56, 3.45)	3.34 (2.86, 3.82)	< *0.001*
LVEF ≥ 50%, n (%)	197 (87.2)	414 (87.3)	0.949	111 (86.7)	102 (82.3)	0.328

*P2Y12 receptor antagonist within 12 months after PCI. *P*, level of statistical significance

*BMI* body mass index, *BP* blood pressure, *MI* myocardial infarction, *PAD* peripheral artery disease, *UAP* unstable angina pectoris, *NSTEMI* non-ST-segment elevation myocardial infarction, *STEMI* ST-elevation myocardial infarction, *HbA1c* Hemoglobin A1c, *TC* total cholesterol, *LDL-C* low-density lipoprotein cholesterol, *TG* triglyceride, *ALT* alanine aminotransferase, *CK-MB* creatine kinase-MB, *NT-proBNP* N-Terminal pro-brain natriuretic peptide, *LVEF* left ventricular ejection fraction, *ACEI* angiotensin-converting enzyme inhibitors, *ARB* angiotensin receptor blockers

Between the very elderly female PCI and medical groups, there were no significant differences in age, BMI, heart rate, systolic/diastolic blood pressure at admission, and also in the medical history of hypertension, diabetes mellitus, dyslipidemia, prior MI and stroke. When compared to medical group, the proportions of STEMI (28.4% vs. 10.1%, *P* < 0.001) and smoking (9.9% vs. 5.8%, *P* = 0.046) were much higher in female PCI group, and the rate of UAP was much lower (47.8% vs. 69%, *P* < 0.001). In laboratory findings, higher levels of LDL-C (*P* = 0.012), ALT (*P* = 0.024) and CK-MB (*P* = 0.002) and lower level of creatinine (*P* = 0.016) were detected in female PCI group. Notably, there were no significant differences in length of stay and log [NT-proBNP] between the two groups. After PS matching, the differences in baseline data were disappeared, except for log [NT-proBNP] (*P* < 0.001), which was significantly increased in the medical group.

### Primary and secondary endpoints in female PCI and medical groups

**Fig. 2:**
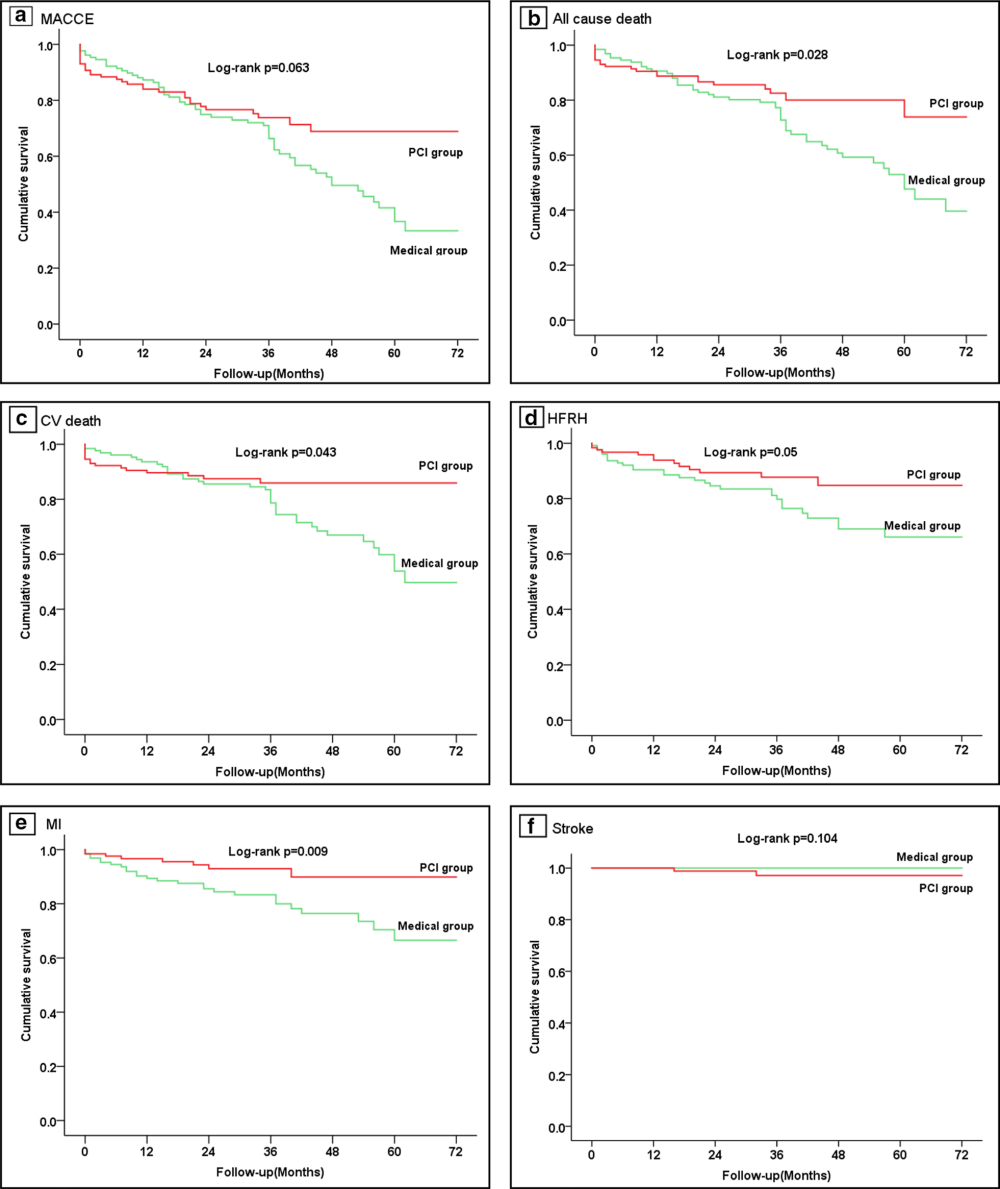
Kaplan–Meier curve analyses for primary and secondary endpoints after PS matching in PCI and medical group. *MACCE* major adverse cardiovascular and cerebrovascular events, *CV* cardiovascular, *HFRH* HF requiring hospitalization, *MI* myocardial infarction

Before PS matching, the risks of CV death (10.8% vs. 18.1%, *P* = 0.011), all-cause death (16.4% vs. 24.3%, *P* = 0.015) and HFRH (9.1% vs. 17.5%, *P* = 0.003) were much lower in female PCI group than that in medical group. But there were no significant differences in primary endpoint (composite MACCE, 24.1% vs. 29.6%, *P* = 0.127) and non-fatal MI (8.2% vs. 10.9%, *P* = 0.262) during the follow-up period of 36 (IQR 23, 48) months. After PS matching, significant differences in primary and most of secondary endpoints were detected between female PCI group (n = 129) and medical group (n = 129). Besides the differences in CV death (12.4% vs. 28.7%, *P* < 0.001), all-cause death (16.3% vs. 37.2%, *P* < 0.001) and HFRH (10.9% vs. 22.5%, *P* = 0.012), the incidences of composite MACCE (24.0% vs. 44.2%, *P* < 0.001), non-fatal MI (6.2% vs. 20.2%, *P* < 0.001) were significantly declined in the female PCI group (Table 2), compared to the female medical group. Kaplan–Meier curves illustrated the incidences of primary and secondary endpoints of the two groups in details (Fig. 2a–f).

**Table 2:** The comparison of MACCE in female PCI and medical group before and after PS matching

Variables	Before PS match		*P* value	After PS match		*P* value
	PCI group (n = 232)	Medical group (n = 497)		PCI group (n = 129)	Medical group (n = 129)	
Composite MACCE, n (%)	56 (24.1)	147 (29.6)	0.127	31 (24.0)	57 (44.2)	< *0.001*
Non-fatal MI, n (%)	19 (8.2)	54 (10.9)	0.262	8 (6.2)	26 (20.2)	*0.001*
Stroke, n (%)	8 (3.4)	6 (1.2)	0.077^*^	2 (1.6)	0 (0)	0.498^*^
HFRH, n (%)	21 (9.1)	87 (17.5)	*0.003*	14 (10.9)	29 (22.5)	*0.012*
CV death, n (%)	25 (10.8)	90 (18.1)	*0.011*	16 (12.4)	37 (28.7)	< *0.001*
All-cause death, n (%)	38 (16.4)	121 (24.3)	*0.015*	21 (16.3)	48 (37.2)	< *0.001*

*Fisher’s exact test. *P*, level of statistical significance

*MACCE* major adverse cardiovascular and cerebrovascular events, *MI* myocardial infarction, *HFRH* HF requiring hospitalization, *CV* cardiovascular

**Fig. 3:**
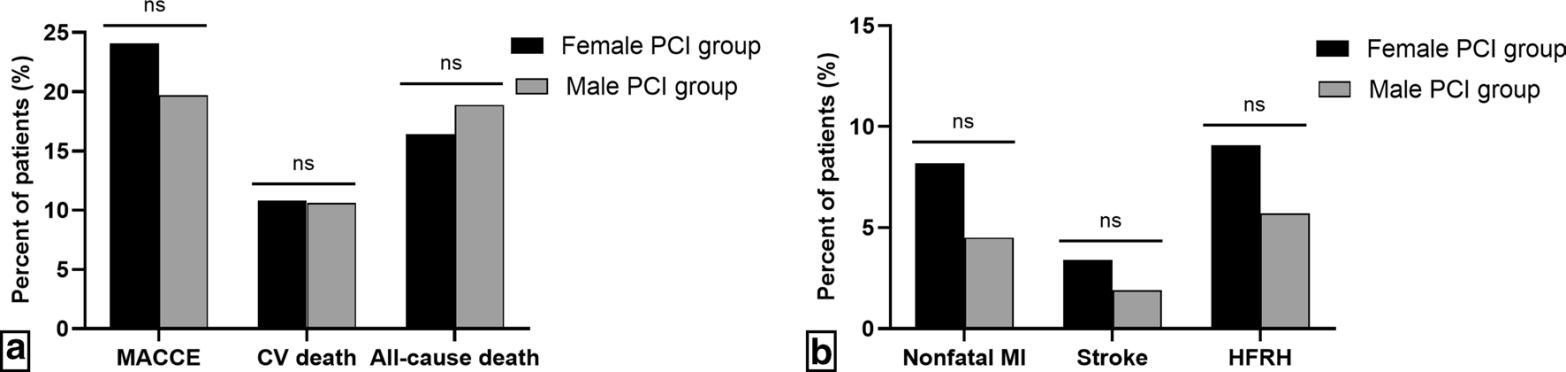
Comparison of MACCE between very elderly female and male PCI patients. *MACCE* major adverse cardiovascular and cerebrovascular events, *CV* cardiovascular, *MI* myocardial infarction, *HFRH* heart failure requiring hospitalization, *PCI* percutaneous coronary intervention

As shown in Fig. 3a, b, the incidences of MACCE were also compared between very elderly female and male PCI group (ACS, ≥ 80 years, n = 264) hospitalized at the same period. The occurrences of composite MACCE (24.1% vs. 19.7%, *P* = 0.232), non-fatal MI (8.2% vs. 4.5%, *P* = 0.094) and HFRH (9.1% vs. 5.7%, *P* = 0.149) tended to be higher in female PCI group, but there were no significant differences between the two groups. Also, there were no remarkable differences in CV death (10.8% vs. 10.6%, *P* = 0.951) and all-cause death (17.7% vs. 18.9%, *P* = 0.456) between the very elderly female and male PCI cohorts.

### Characteristics of the very elderly female PCI patients

Among the very elderly female PCI patients, 24% (n = 56) had MACCE during the follow-up period. In the female PCI patients with MACCE, the proportions of STEMI (44.6% vs. 23.3%, *P* = 0.002) and primary PCI (41.1% vs. 16.5%, *P* < 0.001) were much higher than that in PCI patients without MACCE. The median time of door-to-balloon (D-to-B) was 111 min (72.25, 147.5 min), and there was no significant difference in this time between the female PCI patients with and without MACCE. In addition, compared to female PCI patients without MACCE, the levels of creatinine (81.3 vs. 74.2 umol/L, *P* = 0.029) and log NT-proBNP (3.23 vs. 2.83, *P* < 0.001) were higher and the rate of LVEF ≥ 50% (72.2% vs. 91.9%, *P* < 0.001) was lower in female PCI patients with MACCE. Also, the risk of TIMI major bleeding was much higher in female PCI patients with MACCE (12.5% vs. 4.5%, *P* = 0.035). As for medication during follow-up, female PCI patients with MACCE had much lower proportion of uses in Aspirin (71.4% vs. 84.7%, *P* = 0.026), P2Y12 receptor antagonist (80.4% vs. 96.6%, *P* < 0.001) and β-blocker (42.9% vs. 65.9%, *P* = 0.002) than that in PCI patients without MACCE (Table 3).

**Table 3:** Clinical characteristics of patients with and without MACCE in female PCI group

Variables	MACCE group (n = 56)	Without MACCE group (n = 176)	*P* value
Demographic			
Age (years)	83 (81, 84)	82 (81, 84)	0.188
BMI (kg/m^2^)	24.3 (22.5, 26.5)	24.5 (22.3, 27.0)	0.461
Past medical history			
Hypertension, n (%)	45 (80.4)	140 (79.5)	0.895
Diabetes mellitus, n (%)	18 (32.1)	74 (42.0)	0.187
Dyslipidemia, n (%)	21 (37.5)	75 (42.6)	0.499
Prior MI, n (%)	2 (3.6)	15 (8.5)	0.216
Prior stroke, n (%)	13 (23.2)	43 (24.4)	0.853
Smoking, n (%)	4 (7.1)	19 (10.8)	0.426
Clinical diagnosis			
UAP, n (%)	15 (26.8)	96 (54.5)	< *0.001*
Non-STEMI, n (%)	16 (29.1)	39 (22.2)	0.326
STEMI, n (%)	25 (44.6)	41 (23.3)	*0.002*
Length of stay (days)	8 (6, 10)	7 (6, 10)	0.513
Laboratory finding			
HbA1c (%)	6.2 (5.6, 6.9)	6.2 (5.7, 7.3)	0.308
TC (mmol/L)	4.2 (3.6, 5.3)	4.4 (3.7, 5.1)	0.589
LDL-C (mmol/L)	2.40 (1.88, 3.24)	2.49 (2.02, 2.94)	0.738
TG (mmol/L)	1.3 (0.92, 1.84)	1.3 (0.98, 1.89)	0.555
Creatinine (umol/L)	81.3 (71, 101.6)	74.2 (65.8, 89.1)	*0.029*
ALT (U/L)	18 (10, 31)	14 (10, 20)	*0.036*
CK-MB (ng/ml)	2.1 (1.1, 8.1)	1.7 (1.0, 3.7)	0.071
Log NT-proBNP	3.23 (2.87, 3.71)	2.83 (2.47, 3.30)	< *0.001*
LVEF ≥ 50%, n (%)	39 (72.2)	158 (91.9)	< *0.001*
TIMI bleeding, n (%)			
Major	7 (12.5)	8 (4.5)	*0.035*
Non-major	6 (10.7)	30 (17)	0.254
Medication during follow up, n (%)			
Aspirin	40 (71.4)	149 (84.7)	*0.026*
P2Y12 receptor antagonist^*^	45 (80.4)	170 (96.6)	< *0.001*
β-blocker	24 (42.9)	116 (65.9)	*0.002*
ACEI/ARB	23 (41.1)	89 (50.6)	0.215
Statin	43 (76.8)	153 (86.9)	0.068

*P2Y12 receptor antagonist within 12 months after PCI. *P*, level of statistical significance

*BMI* body mass index, *MI* myocardial infarction, *UAP* unstable angina pectoris, *NSTEMI* non-ST-segment elevation myocardial infarction, *STEMI* ST-elevation myocardial infarction, *HbA1c* Hemoglobin A1c, *TC* total cholesterol, *LDL-C* low-density lipoprotein cholesterol, *TG* triglyceride, ALT alanine aminotransferase, *CK-MB* creatine kinase-MB, *NT-proBNP* N-Terminal pro-brain natriuretic peptide, *LVEF* left ventricular ejection fraction, *ACEI* angiotensin-converting enzyme inhibitors, *ARB* angiotensin receptor blockers

In this female PCI cohort, severe coronary stenosis was found in 99.1% (n = 230) of patients, and three-vessel lesion and CTO were detected in 87.1% (n = 202) and 10.8% (n = 25) of patients, respectively (Table 4). There were no significant differences in the proportions of severe coronary stenosis (99.1% vs. 98.2%, *P* = 0.425), three-vessel lesion (87.5% vs. 86.9%, *P* = 0.991) and CTO (10.7% vs. 10.8%, *P* = 0.986) between female PCI patients with and without MACCE. But pre-procedural TIMI 3 flow was less found in female PCI patients with MACCE (55.4% vs. 78.8%, *P* < 0.001), when compared with PCI patients without MACCE.

**Table 4:** Coronary artery characteristics of patients in female PCI group

Variables	MACCE group n = 56	Without MACCE group n = 176	*P* value
Primary PCI, n (PPCI, %)	23 (41.1)	29 (16.5)	< *0.001*
Door-to-balloon time (PPCI) (min)	112 (78.5,124)	100 (70,188)	0.985
Three-vessel lesion, n (%)	49 (87.5)	153 (86.9)	0.991
Severe stenosis, n (%)	55 (98.2)	175 (99.4)	0.425
CTO rate, n (%)	6 (10.7)	19 (10.8)	0.986
Pre-procedural TIMI 3 flow, n (%)	31 (55.4)	138 (78.4)	< *0.001*
Post-PCI TIMI 3 flow, n (%)	53 (94.6)	173 (98.3)	0.134
Mean stent length (mm)	28 (22,33)	28 (24,33)	0.935
Target vessel-LAD as IRA, n (%)	34 (60.7)	104 (59.1)	0.829
Stent number ≥ 2, n (%)	18 (32.1)	64 (36.4)	0.565
IABP use, n (%)	2 (3.6)	3 (1.7)	0.402
Procedural success rate, n (%)	53 (94.6)	172 (97.7)	0.240

*P*, level of statistical significance

*PCI* percutaneous coronary intervention, *PPCI* primary percutaneous coronary intervention, *CTO* chronic total occlusion, *TIMI* thrombolysis in myocardial infarction, *LAD* left anterior descending coronary artery, *IRA* infarct-related artery, *IABP* intra-aortic balloon pump

All female PCI patients (100%, n = 232) received drug-eluting stent (DES) implantation, and the procedural success rate of PCI was 97% in the very elderly female cohort, as well as 98% in the very elderly male ACS patients in the present study (*P* = 0.605). There were no significant differences in stent number > 2 (32.1% vs. 36.4%, *P* = 0.565) and post-PCI TIMI 3 flow (94.6% vs. 98.3%, *P* = 0.134) between female PCI patients with and without MACCE. Also, there were no significant differences in D-to-B time of primary PCI [112 (78.5, 124 min) vs. 100 (70, 118 min), *P* = 0.985] between the female PCI cohorts with and without MACCE.

### Results of Cox proportional hazards model analyses in female PCI cohort

**Fig. 4:**
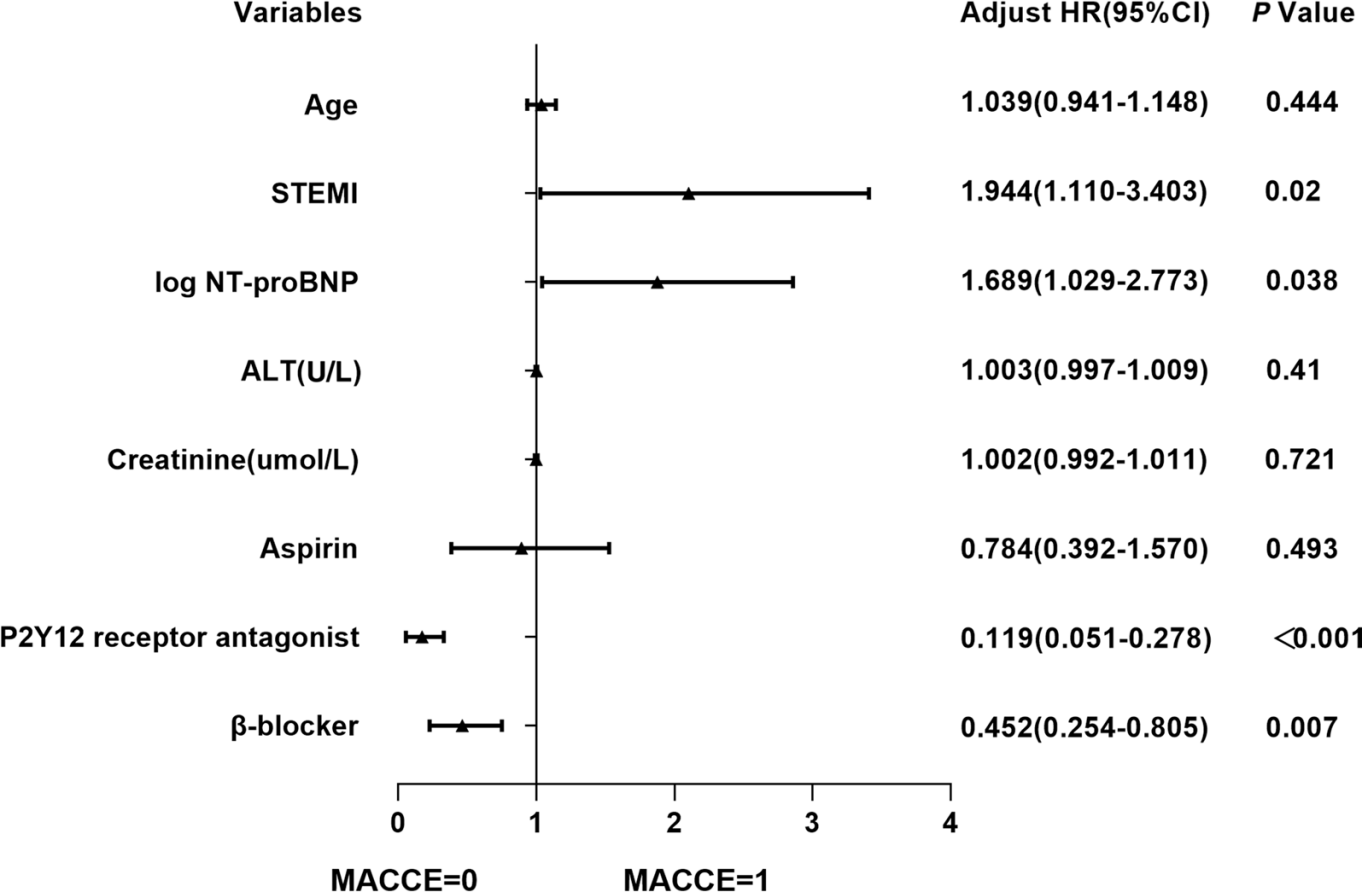
Factors independently associated with composite MACCE in female PCI group in multivariable Cox regression analysis. *STEMI* ST-elevation myocardial infarction, *NT-proBNP* N-Terminal pro-brain natriuretic peptide, *ALT* alanine aminotransferase, *LVEF* left ventricular ejection fraction, *PCI* percutaneous coronary intervention

All the baseline variables (except for variables with statistical collinearity) were included in univariate Cox proportional hazards model to identify the possible predictors for occurrence of MACCE in the female PCI cohort. As detailed in Table 5, ten factors were detected by univariate regression (*P* < 0.05), including age, STEMI, log NT-proBNP, ALT, creatinine, LVEF ≥ 50%, primary PCI, Aspirin, P2Y12 receptor antagonist, β-blocker. Further multivariate Cox regression suggested STEMI (HR 1.944, 95% CI 1.11–3.403, *P* = 0.02) and elevated log NT-proBNP value (HR 1.689, 95% CI 1.029–2.773, *P* = 0.038) were independently associated with the risk of composite MACCE, as well as adherence to P2Y12 receptor antagonist (HR 0.119, 95% CI 0.051–0.278, *P* < 0.001) and β-blocker (HR 0.452, 95% CI 0.254–0.805, *P* = 0.007) medications might help to decrease composite MACCE in the very elderly female PCI patients (Table 5, Fig. 4).

**Table 5:** Cox proportional hazards regression analyses for composite MACCE in female PCI group

Variables	Univariate regression			Multivariate regression		
	HR	95% CI	*P* value	HR	95% CI	*P* value
Age	1.137	1.031–1.253	*0.01*	1.039	0.941–1.148	0.444
STEMI	2.310	1.361–3.919	*0.002*	1.944	1.11–3.403	*0.02*
log NT-proBNP	2.073	1.339–3.211	*0.001*	1.689	1.029–2.773	*0.038*
ALT (U/L)	1.009	1.002–1.015	*0.006*	1.003	0.997–1.009	0.410
Creatinine (umol/L)	1.009	1–1.017	*0.048*	1.002	0.992–1.011	0.721
LVEF ≥ 50%	0.233	0.128–0.426	< *0.001*			
Primary PCI	2.780	1.632–4.737	< *0.001*			
Aspirin	0.492	0.275–0.878	*0.016*	0.784	0.392–1.57	0.493
P2Y12 receptor antagonist	0.147	0.075–0.288	< *0.001*	0.119	0.051–0.278	< *0.001*
β-blocker	0.422	0.248–0.718	*0.001*	0.452	0.254–0.805	*0.007*

*P*, level of statistical significance

*STEMI* ST-elevation myocardial infarction, *NT-proBNP* N-Terminal pro-brain natriuretic peptide, *ALT* alanine aminotransferase, *LVEF* left ventricular ejection fraction, *PCI* percutaneous coronary intervention

## Discussion

We investigated the efficacy and safety of PCI treatment in the very elderly women with ACS in the present study. Our study demonstrated that PCI procedure significantly attenuated the risk of MACCE (24.0% vs. 44.2%, *P* < 0.001), and improved the long-term clinical outcomes in the very elderly female ACS population. In addition, STEMI (HR 1.944, 95% CI 1.11–3.403, *P* = 0.02) and elevated Log- NT-proBNP value (HR 1.689, 95% CI 1.029–2.773, *P* = 0.038) were independently associated with the incidence of MACCE after PCI in this cohort.

Very elderly ACS patients had been historically underrepresented in clinical trials [16, 22]. Limited studies had revealed a larger early hazard and less long-term benefit in the elderly female PCI population, including higher rates of MACCE, mortality and bleeding complication [23, 24]. However, other study determined that coronary revascularization in elderly female ACS patients was associated with lower in-hospital MACCE and 1-year mortality, compared to no revascularized strategy [13]. We found that long-term clinical outcomes in the very elderly female PCI patients were distinctly superior to that in the patients with medical-only treatment during the median follow-up period of 36-month in the study. Compared to medical therapy alone, PCI procedure significantly alleviated the risks of MACCE in the very elderly female ACS cohort (*P* < 0.001), involving composite MACCE, non-fatal MI, HFRH (*P* = 0.012), CV death and all-cause death. Notably, PCI treatment did not extend the length of hospitalization. In earlier studies [15, 25], there was always a lower DES use in elderly female ACS patients compared to the male patients, which might partly contribute to the poor clinical outcomes in this population. All the very elderly female patients received DES implantation in our study, and it was demonstrated that the use of DES in female ACS patients was more effective and safer than bare-metal stent (BMS) during long-term follow-up [26].

On the other hand, the effectiveness and benefits of PCI procedure were comparable between the very elderly female and male ACS patients (≥ 80 years), hospitalized in the same period in our study. Although the incidences of MACCE tended to be higher in female PCI group, there were no significant differences in primary and secondary endpoints between the very elderly female and male ACS patients, such as composite MACCE (*P* = 0.232), non-fatal MI (*P* = 0.094), HFRH (*P* = 0.149), CV death (*P* = 0.951) and all-cause death (*P* = 0.456). Based on the above results, we would like to suggest that very elderly female patients with ACS should be considered for more aggressive PCI strategy in the era of drug-eluting stents.

Very elderly patients always had more complex coronary artery lesions [27], including higher prevalence of multi-vessel disease, calcified lesions, tortuous lesions, and ostial lesions. We found that the coronary artery lesions were generally severe in this very elderly female cohort, but procedural success rate of PCI was relatively satisfactory, which was similar to previous studies of elderly patients (96–98%) [27, 28]. Furthermore, the extent of coronary lesion in female PCI patients with MACCE was similar to that in female PCI patients without MACCE, and there were no significant differences in procedural success rate and number of stents implantation. But the study revealed that female PCI patients with MACCE had significantly lower proportion of pre-procedural TIMI 3 flow (*P* < 0.001), which meant the occurrence of AMI. In female patients with primary PCI, there was no significant difference in median time of door-to-balloon between patients with and without MACCE, which may be attributed to the fact that D-to-B time was relatively shorter in both groups, less than 3 h. Further investigations displayed that STEMI (*P* = 0.002) and primary PCI (*P* < 0.001), worse cardiac (log NT-proBNP, *P* < 0.001; LVEF, *P* < 0.001) and renal function (creatinine level, *P* = 0.029) were associated with the risk of MACCE in female PCI patients. In addition, female PCI patients with MACCE had a higher prevalence of TIMI major bleeding (*P* = 0.035), which might contribute to the occurrence of MACCE in the female PCI cohort. Multivariate Cox regression analysis suggested STEMI (HR 1.944, 95% CI 1.11–3.403, *P* = 0.02) and elevated log- NT-proBNP value (HR 1.689, 95% CI 1.029–2.773, *P* = 0.038) predicted the risk of MACCE in female PCI patients, as well as adherence to P2Y12 receptor antagonist (HR 0.119, 95% CI 0.051–0.278, *P* < 0.001) and β-blocker (HR 0.452, 95% CI 0.254–0.805, *P* = 0.007) medications might help to decrease the risk of MACCE in the very elderly female PCI patients. It highlighted the importance of medication of secondary prevention, which played a key role in the MACCE-free survival during follow-up period.

In clinical practice, there may be some factors that influence cardiologist's decision of PCI strategy. In the present study, more STEMI patients received PCI procedure, and patients with lower creatinine levels were more likely to experience PCI treatment. In addition, NT-proBNP was another important factor considered in PCI decision.

### Limitations

The main limitation of the study is the retrospective design, single-centre data and experience. So, decision of therapy strategy reflected the convention and tendency of our single center, which may affect the objectivity of the conclusion. Furthermore, the sample size was relatively small after propensity matching (n = 129), especially the cases of female PCI patients with MACCE (n = 56), which may have some effect on the final clinical outcome analysis. We look forward to further studies with larger sample size, or a multicenter, prospective design to provide more information and understanding in this field.

## Conclusion

In summary, PCI procedure attenuated the risks of MACCE and improved the long-term clinical outcomes in the very elderly female ACS patients in the era of drug-eluting stents. Aggressive PCI strategy should be considered as a treatment option in this population, and attentions should be paid to some factors that might have impacts on the clinical prognosis after PCI.

## Data Availability

The datasets used and/or analyzed during the current study are available from the corresponding author on reasonable request.

## Abbreviations

MACCE: Major adverse cardiovascular and cerebral event
PCI: Percutaneous coronary intervention
PPCI: Primary percutaneous coronary intervention
ACS: Acute coronary syndrome
MI: Myocardial infarction
HFRH: Heart failure requiring hospitalization
CV: Cardiovascular
STEMI: ST-segment elevation myocardial infarction
NT-proBNP: N-Terminal pro-brain natriuretic peptide
NSTEMI: Non-ST-segment elevation myocardial infarction
UAP: Unstable angina pectoris
ECG: Electrocardiogram
ALT: Alanine aminotransferase
CK-MB: Creatine kinase-MB
ECHO: Echocardiography
LVEF: Left ventricular ejection fraction
HbA1c: Hemoglobin A1c
TC: Total cholesterol
LDL-C: Low-density lipoprotein cholesterol
TG: Triglyceride
TIMI: Thrombolysis in myocardial infarction
LAD: Left atrium diameter
HR: Hazard ratios
CI: Confidence intervals
BMI: Body mass index
BP: Blood pressures
IABP: Intra-aortic balloon pump
ACEI: Angiotensin-converting enzyme inhibitors
ARB: Angiotensin receptor blockers
DES: Drug-eluting stent
D-to-B: Door-to-balloon
